# Association Between Serum Homocysteine and Chronic Kidney Disease Severity in Vietnamese Outpatients With Type 2 Diabetes: A Cross‐Sectional Study

**DOI:** 10.1002/edm2.70339

**Published:** 2026-09-16

**Authors:** Loi Ngoc Ho, Tien Van Tran, Tinh Trung Truong Tran, Nhan Trong Le, Loc Huu Quach, Khanh Minh Thanh, Hien Ngoc Tran, Hang Khanh Hoang, Ha Hai Tran, Tuan Quoc Le

**Affiliations:** ^1^ University Medical Center Ho Chi Minh City Ho Chi Minh City Vietnam; ^2^ University of Medicine and Pharmacy at Ho Chi Minh City Ho Chi Minh City Vietnam; ^3^ Hospital for Rehabilitation—Professional Disease Ho Chi Minh City Vietnam; ^4^ Faculty of Medicine Hong Bang International University; ^5^ Faculty of Medicine Van Lang University Ho Chi Minh City Vietnam; ^6^ Buon Ma Thuot Medical University Buon Ma Thuot Vietnam; ^7^ Tra Vinh University Vinh Long Vietnam

**Keywords:** chronic kidney disease, CKD severity, homocysteine, Type 2 diabetes mellitus, Vietnamese

## Abstract

**Introduction:**

CKD is a major complication of T2DM and a leading cause of end‐stage kidney disease. Because urinary albumin‐to‐creatinine ratio (UACR) and eGFR have recognized limitations, additional biomarkers may help characterize kidney dysfunction. HCY is frequently elevated in CKD and may reflect pathways of renal injury. We evaluated the association of HCY with kidney dysfunction and its cross‐sectional discrimination of CKD status in Vietnamese adults with T2DM.

**Methods:**

A descriptive cross‐sectional study was conducted among adult outpatients with T2DM recruited at UMC HCMC (Campus 2) from December 2023 to December 2025 using convenience sampling. CKD was defined as eGFR < 60 mL/min/1.73 m^2^ and/or UACR > 30 mg/g. HCY and other laboratory parameters were measured using standardized protocols; eGFR was calculated using the 2021 CKD‐EPI creatinine‐cystatin C equation. Correlation, logistic regression, and ROC analyses were performed. Albuminuria alone was examined in a sensitivity analysis, and AUCs were compared using DeLong's test.

**Results:**

Among 201 participants, 164 (81.6%) met the study‐defined CKD criteria. HCY was higher in participants with CKD and increased across worsening eGFR and albuminuria categories. HCY correlated more strongly with creatinine and cystatin C (*r* = 0.67 for both) and eGFR (*r* = −0.59) than with UACR (*r* = 0.26). Higher HCY remained associated with CKD after adjustment for age and HbA1c (adjusted OR = 1.27, 95% CI 1.14–1.42). The HCY‐only AUC was 0.79; the Youden‐derived threshold was 11.35 μmol/L (sensitivity 0.76; specificity 0.78). For albuminuria alone, the association persisted (OR = 1.08, 95% CI 1.03–1.14), but discrimination was modest (AUC = 0.63). The multivariable AUC was 0.812 and did not differ significantly from the HCY‐only AUC (DeLong *p* = 0.325).

**Conclusions:**

Higher HCY was associated with CKD and kidney dysfunction, with a stronger relationship to filtration markers than to albuminuria. These cross‐sectional findings do not establish screening or prognostic utility. The 11.35 μmol/L threshold is exploratory and requires external validation.

## Graphical abstract

In 201 Vietnamese adults with Type 2 diabetes, higher serum homocysteine was associated with CKD and showed a stronger relationship with filtration markers than with albuminuria. Its discrimination of CKD status was moderate‐to‐good (AUC 0.79), while the 11.35 μmol/L threshold requires external validation.

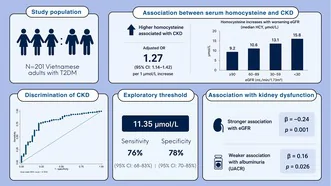

## 1 Introduction

In the context of the rapidly escalating burden of Type 2 Diabetes Mellitus (T2DM), chronic kidney disease (CKD) has emerged as a formidable public health challenge, accounting for up to one‐half of end‐stage kidney disease cases. Although albuminuria and the estimated glomerular filtration rate (eGFR) remain the principal markers for CKD assessment, both have recognized limitations in clinical practice. Albuminuria may vary according to clinical conditions, whereas reductions in eGFR may become apparent only after a degree of kidney dysfunction has developed [1]. These limitations have prompted interest in additional biomarkers that may complement conventional assessment of kidney dysfunction in patients with T2DM.

Homocysteine (HCY) has attracted increasing attention as a potential link within the complex pathophysiology of renal injury, extending beyond its role as an intermediate in methionine metabolism. Accumulating experimental and observational evidence suggests that elevated HCY may be linked to renal injury through multiple interacting mechanisms. Specifically, HCY has been reported to promote oxidative stress and inflammatory responses by increasing reactive oxygen species production, potentially affecting endothelial cells of the renal microvasculature [2]. In parallel, HCY may interfere with DNA methylation, inducing epigenetic alterations in gene regulation that could modify renal parenchymal structure and function in the setting of chronic hyperglycemia [3]. Moreover, experimental studies have implicated elevated HCY in pathways related to glomerulosclerosis [4].

Multiple studies have reported that HCY levels are frequently elevated in patients with diabetic CKD. Prior evidence also linked elevated HCY to renal injury‐related pathways in individuals with T2DM, including inflammation, oxidative stress, and altered DNA methylation [5]. However, these findings do not establish whether elevated HCY is a cause or consequence of kidney dysfunction.

Despite existing research on HCY and CKD in patients with T2DM, studies examining the association between HCY and kidney dysfunction among Vietnamese adults with T2DM remain limited [6]. Notably, the discriminative characteristics of HCY for CKD status in this population have not been clearly established [7, 8].

Therefore, we conducted this study with the following objectives: (1) to determine the association between serum HCY concentration and renal function in patients with T2DM; and (2) to evaluate the discriminative ability of serum HCY for CKD status in this outpatient cohort.

## 2 Materials and Methods

### 2.1 Subjects

This descriptive cross‐sectional study was conducted using a convenience sample of adult patients with T2DM recruited from the Outpatient Department of University Medical Center Ho Chi Minh City (UMC HCMC), Branch 2, between December 2023 and December 2025.

#### 2.1.1 Inclusion and Exclusion Criteria

Inclusion Criteria

- Patients diagnosed with T2DM according to the ADA 2023 criteria [9].
- Age ≥ 18 years.
- Provided written informed consent and voluntarily agreed to participate.

Exclusion Criteria:

- Presence of acute kidney injury.
- Presence of an acute infection.
- Use of medications known to affect serum HCY levels (e.g., vitamin B3, methotrexate, and antiepileptic drugs such as phenytoin or carbamazepine).
- Presence of active liver disease or malignancy.

### 2.2 Sample Size and Sampling

Objective 1: To determine the association between serum HCY concentration and renal function in patients with T2DM. Formula: Sample size estimation for a correlation coefficient.

$$n\geq {\left(\frac{Z_{1-\frac{\alpha }{2}}+Z_{1-\beta }}{0.5\;\ln\left(\frac{1+r}{1-r}\right)}\right)}^{2}+3$$

where $Z_{1-\alpha /2}=1.96$; $Z_{1-\beta }=0.8416$; r=−0.581(based on Juan Wu et al. [10]); n≥21 patients.

Objective 2: To evaluate the discriminative performance of serum HCY for CKD status in patients with T2DM.

Formula: Sample size estimation for the area under the receiver operating characteristic curve (AUC).

$$n_{\mathrm{CKD}}=n_{\text{non}-\mathrm{CKD}}\geq \frac{Z_{1-\frac{\alpha }{2}}^{2}\;V_{\mathrm{AUC}}}{d^{2}}\;V_{\mathrm{AUC}}=\left(0.0099\times e^{-\frac{a^{2}}{2}}\right)\left(6a^{2}+16\right)\;a=1.414\times Z_{\mathrm{AUC}}$$

where $Z_{1-\alpha /2}=1.96$; $Z_{1-\beta }=0.8416$; d=0.1;AUC=0.905(based on Maria Petrovna Kruglova et al. [11]); $n_{\mathrm{CKD}}=n_{\text{non}-\mathrm{CKD}}\geq 26$ patients.

In practice, we enrolled a total of 201 patients with T2DM, including 37 participants without CKD and 164 participants who met the study‐defined CKD criteria.

### 2.3 Data Collection

Demographic characteristics (age, sex) and clinical information (BMI, systolic blood pressure, and diastolic blood pressure) were extracted from medical records. Participants provided a fasting venous blood sample after 8–12 h of fasting and an early‐morning random urine specimen. Laboratory measurements, including HCY, creatinine, cystatin C, FPG, HbA1c, and UACR, were performed in the same laboratory using standardized procedures.

For this cross‐sectional analysis, CKD status (yes/no) was operationally defined as an eGFR < 60 mL/min/1.73 m^2^ and/or a UACR > 30 mg/g in patients with T2DM. Kidney dysfunction was categorized by eGFR and UACR. By eGFR, participants were classified as G1–G2 (≥ 60 mL/min/1.73 m^2^), G3 (30–59 mL/min/1.73 m^2^), or G4–G5 (< 30 mL/min/1.73 m^2^). By UACR, albuminuria was classified as A1 (< 30 mg/g), A2 (30–300 mg/g), or A3 (> 300 mg/g).

All assays were conducted in a single laboratory according to standardized protocols. Serum HCY was quantified using a chemiluminescent immunoassay on the ADVIA Centaur XP system (μmol/L). Serum creatinine was measured using an enzymatic method on the AU5800 Beckman Coulter analyser (mg/dL), whereas cystatin C was quantified by immunoturbidimetry on the ALINITY system (mg/L). eGFR was estimated using the 2021 CKD‐EPI creatinine–cystatin C equation (mL/min/1.73 m^2^) [12]. UACR was determined from urine samples: urinary albumin was measured by immunoturbidimetry and urinary creatinine by an enzymatic method on the AU5800 Beckman Coulter analyser, and results were expressed as mg/g. HbA1c was measured by high‐performance liquid chromatography (HPLC) on the Bio‐Rad D‐10 system (%), and FPG was quantified using the hexokinase method on the Roche Cobas 6000 system (mg/dL).

### 2.4 Statistical Analysis

Data were initially entered and stored in Microsoft Excel 2019 and subsequently analysed using STATA MP 17.0. The Kolmogorov–Smirnov test was applied to assess data distribution. Categorical variables were reported as counts and percentages, whereas continuous variables were summarized as mean ± standard deviation for normally distributed data or as median and interquartile range for skewed data. Statistical significance was defined as a two‐sided *p* value < 0.05.

To evaluate correlations between continuous variables, Pearson's or Spearman's correlation coefficients were used as appropriate based on distribution. The direction of the association was interpreted according to the sign of the coefficient, with positive values indicating direct correlations and negative values indicating inverse correlations.

Differences in central tendency were assessed using Student's *t*‐test or the Mann–Whitney U test, as appropriate. Binary logistic regression was used to examine the cross‐sectional association between HCY levels and CKD status. Results were presented as odds ratios (ORs) with 95% confidence intervals (CIs). Model fit was assessed using the likelihood ratio test and the pseudo‐$R^{2}$ statistic.

Receiver operating characteristic (ROC) curve analysis was performed to quantify discrimination between participants with and without study‐defined CKD. The area under the curve (AUC), Youden‐derived threshold, sensitivity, and specificity were reported. Because the threshold was derived and evaluated in the same cohort, it was considered exploratory. A nonparametric DeLong test was used to compare the correlated AUCs of the HCY‐only model and the multivariable model.

Because study‐defined CKD status incorporated eGFR calculated from serum creatinine and cystatin C, eGFR, creatinine, and cystatin C were not entered as predictors in the multivariable logistic regression model. The model was adjusted for age and HbA1c. To address the possibility that the observed HCY‐CKD association was predominantly filtration‐related, a sensitivity analysis used albuminuria alone (UACR ≥ 30 mg/g) as the binary outcome, independent of the eGFR criterion; univariable logistic regression and ROC analysis were performed for this outcome.

### 2.5 Ethical Considerations

The research proposal was accepted ethically in research by the Biomedical Research Ethics Committees at the University of Medicine and Pharmacy at Ho Chi Minh City number 1171/HĐĐĐ‐ĐHYD signed on 23/11/2023. The English translation of the ethics approval document and the signed Vietnamese original are provided as Files S1 and S2, respectively. Written informed consent was obtained from all participants.

## 3 Results

**FIGURE 1:**
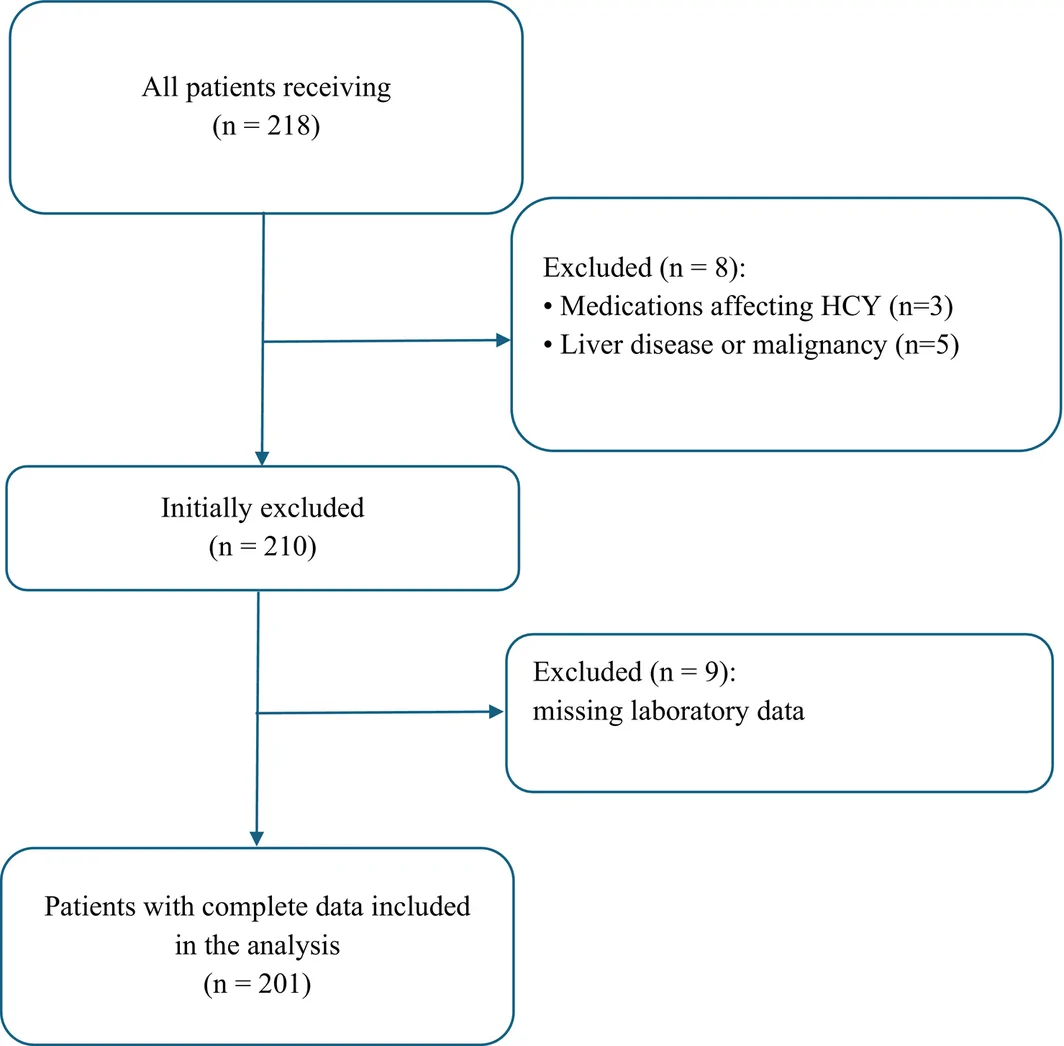
Flowchart of patient selection.

During the study period, 218 patients with T2DM were assessed for eligibility. Of these, eight were excluded because they met exclusion criteria, including use of medications affecting HCY (*n* = 3) and liver disease or malignancy (*n* = 5). An additional nine patients were excluded due to missing laboratory data. No further exclusions were made. The final analytic sample comprised 201 patients (Figure 1) with a male‐to‐female ratio of approximately 3:2 and a mean age of 64.85 ± 10.38 years.

The 201 patients with T2DM were classified into two groups: those with CKD (81.6%) and those without CKD (18.4%). The mean age in the CKD group (65.85 ± 10.58 years) was significantly higher (*p* = 0.004) than that in the non‐CKD group (60.41 ± 8.22 years). No statistically significant differences were observed between groups with respect to sex, BMI, blood pressure, or HbA1c. The CKD group exhibited higher levels of HCY, creatinine, cystatin C, and UACR, whereas eGFR was significantly lower compared with the non‐CKD group (all *p* < 0.001) (Table 1).

**TABLE 1:** Characteristics of patients with T2DM (*n* = 201).

Characteristics	Total	Group 1	Group 2	*p*
	*n* = 201	*n* = 37 (18.41%)	*n* = 164 (81.59%)	
Age (Mean ± SD)	64.85 ± 10.38	60.41 ± 8.22	65.85 ± 10.58	0.004*
Gender: *n* (%)				
Male	82 (40.80%)	13 (35.14%)	69 (42.07%)	0.438***
Female	119 (59.20%)	24 (64.86%)	95 (57.93%)	
BMI (Mean ± SD)	24.11 ± 3.46	24.10 ± 3.65	24.12 ± 3.43	0.979*
SBP (IQR)	134 (120–150)	130 (120–140)	136.50 (120–150.50)	0.157**
DBP (IQR)	80 (73–90)	80 (80–85)	80 (70–90)	0.545**
HbA1c (IQR)	7.60 (6.68–8.90)	7.30 (6.63–8.30)	7.60 (6.69–8.98)	0.310**
HCY (Mean ± SD)	14.96 ± 6.28	10.68 ± 4.09	15.93 ± 6.29	< 0.001*
Cre (IQR)	1.31 (1.05–1.69)	0.98 (0.83–1.06)	1.42 (1.18–1.84)	< 0.001**
Cys (IQR)	1.53 (1.14–1.95)	0.96 (0.88–1.05)	1.69 (1.40–2.05)	< 0.001**
eGFR (Cre + Cys) (Mean ± SD)	50.93 ± 25.18	81.95 ± 15.05	43.93 ± 21.46	< 0.001*
UACR (IQR)	44.30 (11.80–296.70)	10.80 (5.40–15)	77.80 (21.10–514.05)	< 0.001**

Abbreviations: BMI, body mass index; Cre, creatinine; Cys, cystatin C; DBP, diastolic blood pressure; eGFR, estimated glomerular filtration rate; HCY, homocysteine; IQR, interquartile range; SBP, systolic blood pressure; SD, standard deviation; UACR, urinary albumin‐to‐creatinine ratio.

* Independent samples *t*‐test.

** Mann–Whitney *U* test.

*** Chi‐square test.

Serum HCY concentrations increased with greater severity of renal impairment. Stratified by eGFR, HCY rose markedly as kidney function declined (G1–G2: 10.3 ± 3.5; G3: 15.5 ± 5.7; G4–G5: 20.5 ± 5.9 μmol/L), with statistically significant differences across categories (*p* < 0.001). Similarly, stratified by UACR, HCY increased progressively with worsening albuminuria (A1: 13.4 ± 5.1; A2: 15.6 ± 8.1; A3: 16.9 ± 4.8 μmol/L), *p* < 0.001 (Table 2).

**TABLE 2:** Serum HCY levels across categories of kidney dysfunction (*n* = 201).

Category	*N* = 201	HCY	*p*
	*n* (%)	Mean ± SD	
eGFR			< 0.001*
G1–G2	57 (28.4%)	10.3 ± 3.5	
G3	105 (52.2%)	15.5 ± 5.7	
G4–G5	39 (19.4%)	20.5 ± 5.9	
UACR			< 0.001**
A1	88 (43.8%)	13.4 ± 5.1	
A2	63 (31.3%)	15.6 ± 8.1	
A3	50 (24.9%)	16.9 ± 4.8	

Abbreviations: eGFR, estimated glomerular filtration rate; HCY, homocysteine; SD, standard deviation; UACR, urinary albumin‐to‐creatinine ratio.

* One‐way ANOVA.

** Kruskal–Wallis test.

**FIGURE 2:**
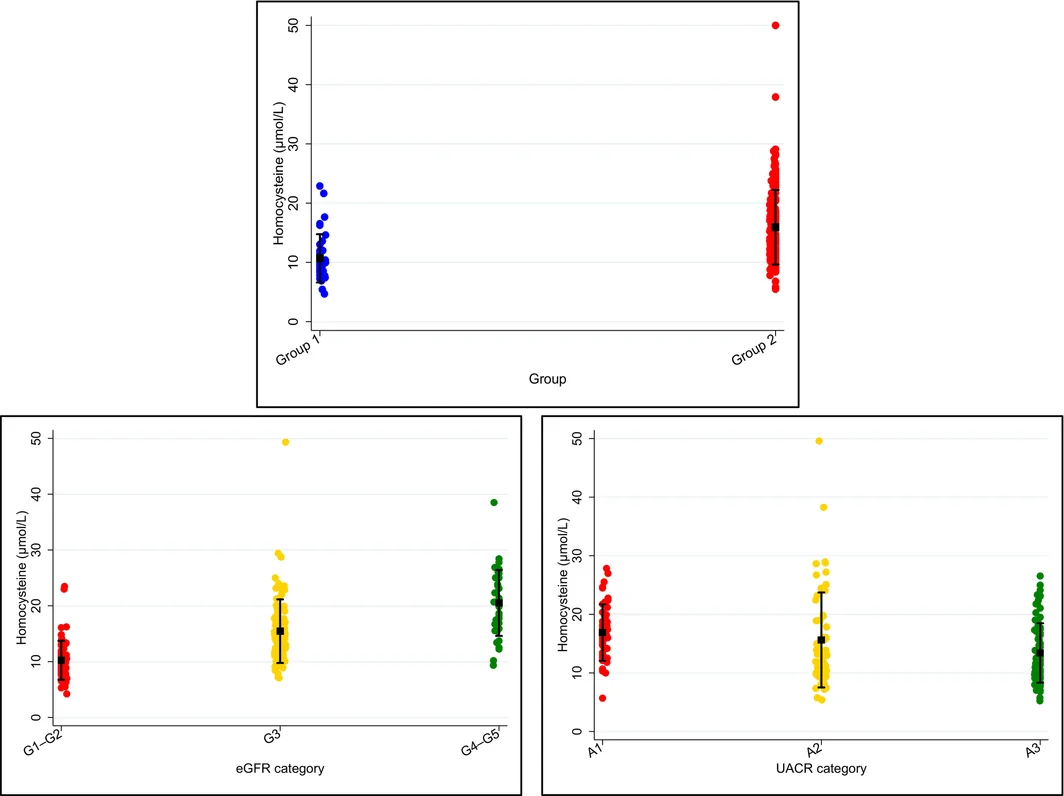
Serum HCY levels across categories of kidney dysfunction.

Serum HCY levels were substantially higher in the renal impairment group (Group 2) than in the no renal impairment group (Group 1), and increased in parallel with CKD severity. Specifically, HCY showed an upward trend with decreasing eGFR (G1–G2 < G3 < G4–G5) and with increasing albuminuria category (A1 < A2 < A3). The distribution of data points also suggests several high‐HCY outliers among patients with more advanced renal impairment (Figure 2).

HCY showed a statistically significant weak positive correlation with age and UACR. In contrast, HCY demonstrated strong positive correlations with creatinine and cystatin C and a strong negative correlation with eGFR; all of these correlations were statistically significant (*p* < 0.001). No statistically significant correlations were observed between HCY and blood pressure or HbA1c (Table 3).

**TABLE 3:** Correlation between HCY and clinical and laboratory characteristics (*n* = 201).

Characteristics	HCY	
	Correlation coefficient	*p*
Age	0.18	0.011**
SBP	0.12	0.099*
DBP	−0.06	0.403*
HbA1c	−0.13	0.062*
Cre	0.67	< 0.001*
Cys	0.67	< 0.001*
eGFR (Cre + Cys)	−0.59	< 0.001**
UACR	0.26	< 0.001*

Abbreviations: Cre, creatinine; Cys, cystatin C; DBP, diastolic blood pressure; eGFR, estimated glomerular filtration rate; HCY, homocysteine; SBP, systolic blood pressure; UACR, urinary albumin‐to‐creatinine ratio.

* Spearman's rank correlation test.

** Pearson's rank correlation test.

Binary logistic regression indicated that for each 1 μmol/L increase in HCY, the odds of CKD increased by 28% among patients with T2DM (*p* < 0.001) (Table 4).

**TABLE 4:** Univariable logistic regression of serum HCY and CKD status in patients with T2DM (*n* = 201).

	OR	SE	*z*	95% CI	*p*
HCY	1.28	0.07	4.43	1.15–1.43	< 0.001
_cons	0.19	0.12	−2.53	0.05–0.69	0.012
LR chi^2^(1) = 30.87; Pseudo *R* ^2^ = 0.16					< 0.001

Abbreviations: CI, Confidence interval; HCY, homocysteine; OR, odds ratios; SE, Standard error.

**FIGURE 3:**
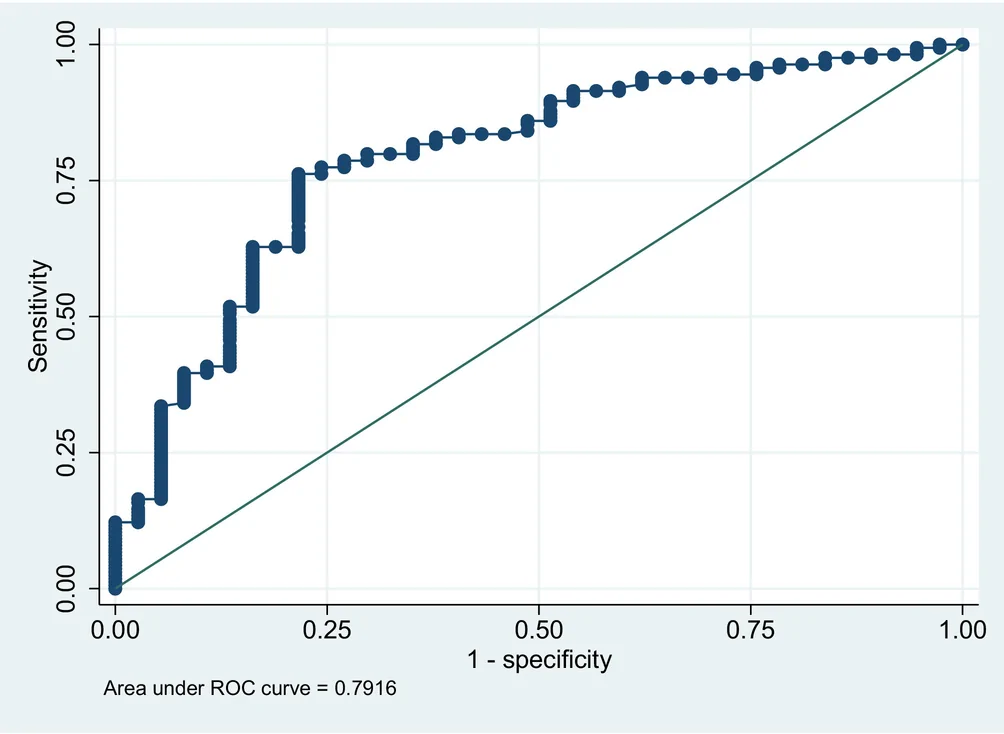
Discriminative ability of serum HCY for CKD status based on ROC curve analysis.

In the univariable analysis of CKD status, the AUC was 79.16% (95% CI: 0.71–0.87; *p* < 0.001), demonstrating discrimination between the two groups within this cohort. The optimal cutoff value was 11.35 μmol/L, yielding a sensitivity of 0.76 and a specificity of 0.78. At this threshold, the mean HCY level in the CKD group lay entirely above the cutoff, whereas most patients without CKD were below it. The ROC curve exhibited a steep initial ascent and a pronounced curvature, suggesting stable discrimination and good overall performance, particularly given the balanced sensitivity and specificity at the 11.35 μmol/L cutoff (Table 5; Figure 3).

**TABLE 5:** ROC characteristics of serum HCY for discriminating CKD status (*n* = 201).

AUC	Cut off threshold	Sensitivity	Specificity	SE	95% CI	*p*
0.7916	11.35	0.76	0.78	0.04	0.71–0.87	< 0.001

Abbreviations: AUC, area under the ROC curve; CI, Confidence interval; SE, Standard error.

In a sensitivity analysis using albuminuria alone (UACR ≥ 30 mg/g) as the outcome, serum HCY remained associated with albuminuria (OR = 1.08, 95% CI: 1.03–1.14; *p* = 0.003). ROC analysis showed modest discrimination (AUC = 0.6278, 95% CI: 0.55–0.71; *p* = 0.0398).

**FIGURE 4:**
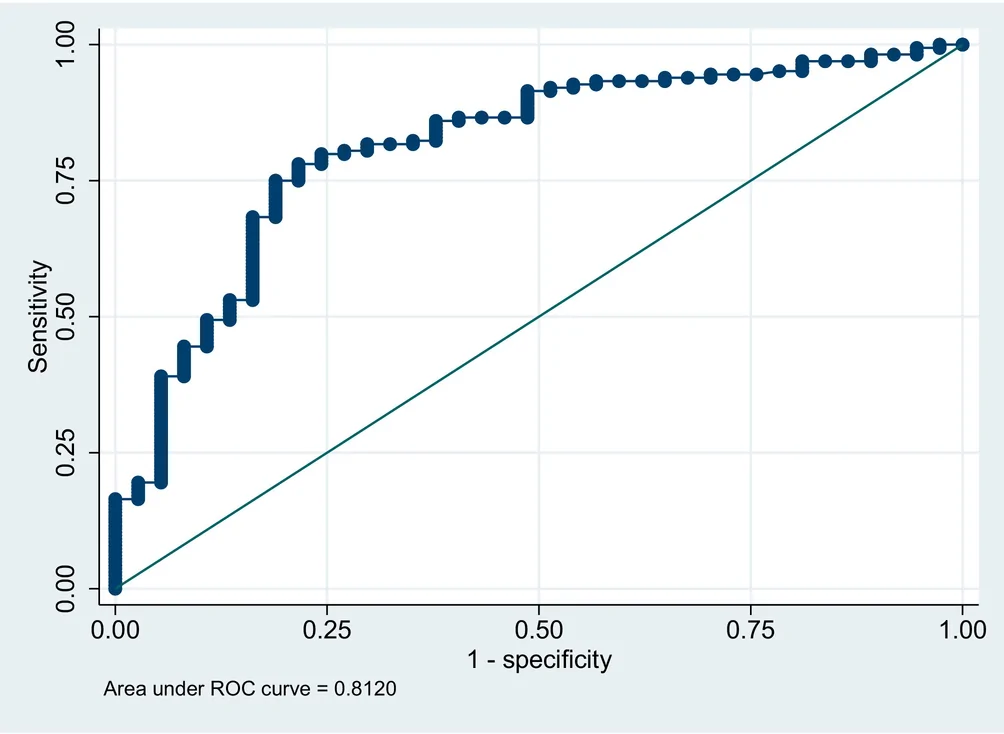
ROC curve for the multivariable model including age, HbA1c, and serum HCY.

The multivariable model for CKD status was statistically significant (*p* < 0.001), with an AUC of approximately 81.2% (95% CI: 0.73–0.89). No multicollinearity was detected among the predictors. The model explained 20.06% of the variability in CKD risk when age, HbA1c, and HCY were included; age and HCY remained associated with CKD status after adjustment. A formal comparison using DeLong's test showed that the AUC of the multivariable model (0.8120) was not significantly different from that of the HCY‐only model (0.7916; χ^2^ = 0.97, *p* = 0.3249) (Table 6; Figure 4).

**TABLE 6:** Multivariable logistic regression model and risk of CKD in T2DM patients (*n* = 201).

	aOR	SE	*z*	VIF	CI 95%	*p*
Age	1.04	0.02	2.01	1.04	1.00–1.09	0.044
HbA1c	1.22	0.13	1.84	1.04	0.99–1.51	0.065
HCY	1.27	0.07	4.29	1.01	1.14–1.42	< 0.001
_cons	0.00	0.01	−3.23		0.00–0.10	0.001
LR chi^2^(3) = 38.51; Pseudo *R* ^2^ = 0.2006						< 0.001
AUC = 0.8120		0.04			0.73–0.89	< 0.001

Abbreviations: aOR, adjusted odds ratios; AUC, area under the ROC curve; CI, confidence interval; HCY, homocysteine; SE, standard error; VIF, variance inflation factor.

## 4 Discussion

In our study, CKD was highly prevalent in our T2DM cohort, and age was the most prominent factor associated with CKD. Patients with CKD were significantly older than those without CKD, whereas sex, BMI, blood pressure, and HbA1c were comparable between groups. This age difference is consistent with the known epidemiology of CKD in T2DM; however, given the cross‐sectional design and the marked group imbalance, it should not be interpreted as evidence of CKD onset or progression [13, 14]. Accordingly, CKD screening and monitoring should be prioritized, particularly in older patients with T2DM. Compared with the non‐CKD group, patients with CKD had higher HCY, creatinine, cystatin C, and UACR levels and lower eGFR, with statistically significant differences (*p* < 0.001). The combination of increased creatinine and UACR with reduced eGFR is characteristic of diabetic kidney disease and reflects both structural injury and impaired filtration. Albuminuria often represents an early manifestation of glomerular damage, whereas declining eGFR reflects progression of renal impairment. Therefore, creatinine, UACR, and eGFR remain the core indices for CKD staging and longitudinal monitoring under current recommendations [15]. Elevated cystatin C in the CKD group further supports reduced filtration and suggests that cystatin C may be a more sensitive filtration marker than creatinine in certain settings, particularly in older adults or when muscle mass varies. This is consistent with the meta‐analysis by Shlipak et al., showing that cystatin C–based eGFR, either alone or combined with creatinine, improves risk stratification and is more strongly associated with adverse outcomes, including mortality and end‐stage kidney disease, than creatinine alone [16]. Beyond conventional CKD markers, we observed higher HCY levels among patients with CKD, indicating an association between HCY and impaired renal function in this cohort. This observation aligns with Shen et al., who reported that hyperhomocysteinemia was associated with older age and poorer renal function among patients with T2DM [17]. Collectively, these findings support an association between HCY and the presence and greater severity of CKD in T2DM.

Our findings indicate that plasma HCY increased stepwise with worsening kidney function, rising as eGFR declined from G1–G2 to G3 and G4–G5 (*p* < 0.001). Notably, variability in HCY was greatest in advanced CKD, suggesting increasing heterogeneity in HCY metabolism and clearance at later stages. This pattern is consistent with Xulei Zheng et al., who reported increasing HCY with CKD progression and proposed HCY > 12 μmol/L as a predictor of diabetic kidney disease progression [18]. It also parallels findings from Shen et al., who observed higher HCY in patients with CKD and a stepwise increase across CKD stages [17]. The greater dispersion of HCY at very low eGFR in our cohort may reflect inter‐individual differences in renal injury severity, compensatory capacity, inflammatory and oxidative stress burden, nutritional status, and broader metabolic disturbances. Taken together, these findings support an association between higher HCY and greater kidney dysfunction in this cohort; whether HCY has prognostic value for CKD progression requires longitudinal evaluation.

Correlation analyses showed that HCY was weakly but significantly correlated with age and UACR, while demonstrating moderate correlations with creatinine and cystatin C and an inverse correlation with eGFR (all *p* < 0.001). The stronger correlations of HCY with creatinine, cystatin C, and eGFR than with UACR suggest that serum HCY in this cohort may predominantly reflect impaired glomerular filtration rather than an independent marker of glomerular injury. In the sensitivity analysis, HCY remained associated with albuminuria alone (OR = 1.08, 95% CI: 1.03–1.14; *p* = 0.003), but the modest AUC (0.628) indicates limited discrimination for albuminuria. Prior studies report similar patterns. Vineet Kumar et al. found an inverse correlation between HCY and eGFR and a positive correlation with HbA1c in T2DM, suggesting links between HCY, glycemic control, and kidney disease progression [19]. A meta‐analysis by Zhu and Fan (2024) reported higher HCY with increasing albuminuria severity and an association between elevated HCY and higher risk of diabetic kidney disease [20, 21], consistent with our observed association between HCY and UACR. In addition, a meta‐analysis by Wei Chen et al. swshowed that elevated HCY was associated with a higher risk of CKD, supporting our observation that HCY increases as eGFR declines [22]. Kruglova et al. further suggested prognostic relevance, reporting that HCY remained the only statistically significant variable in a 5‐year survival model, underscoring its potential as a marker of disease severity and adverse risk in CKD [11]. Mechanistically, the association between HCY and kidney injury may involve multiple pathways, including oxidative stress and reactive oxygen species generation, NF‐κB–mediated inflammation, TGF‐β1 activation with mesangial expansion and fibrosis, and podocyte apoptosis leading to increased glomerular permeability and albuminuria. However, these mechanisms cannot be inferred from the present cross‐sectional data, and the direction of the association between HCY and kidney dysfunction remains uncertain.

HCY also demonstrated discrimination between patients with and without CKD within this cohort (AUC 0.79; *p* < 0.001), with a Youden‐derived exploratory cutoff of 11.35 μmol/L providing balanced sensitivity and specificity. These findings are broadly consistent with prior reports, although performance and thresholds vary by population. Yu‐Lin Shih et al. reported a lower AUC and a higher cutoff in a community‐based middle‐aged to older population [23], while Subhash Chand et al. reported an AUC of 0.73 among patients with T2DM [24]. Kruglova et al. observed stepwise increases in HCY with CKD severity, supporting its potential role in staging [11]. Given the high CKD prevalence and the imbalance between CKD and non‐CKD groups, this cutoff should be regarded as cohort‐specific and exploratory rather than as a validated screening threshold.

The multivariable model including HCY, age, and HbA1c had an AUC of 0.812; however, DeLong's test showed no statistically significant improvement over the HCY‐only model (AUC 0.792; *p* = 0.3249). Therefore, incremental discriminative value cannot be inferred from the present data. Higher HCY remained associated with CKD after adjustment for age and HbA1c. The persistence of HCY in multivariable models reported by Shih et al. and Shen et al. further supports its independent association with CKD risk [17, 20]. Because this study was not specifically designed or adequately powered to evaluate the nonalbuminuric DKD phenotype, we do not infer a specific role for HCY in this subgroup. The cutoff of 11.35 μmol/L should be considered exploratory and requires prospective external validation before clinical implementation.

### 4.1 Limitations of the Study

Although this study provides additional evidence regarding the association between HCY and renal dysfunction in patients with T2DM, several limitations should be acknowledged. First, the cross‐sectional design precludes causal inference; elevated HCY may represent both a consequence of impaired kidney function and a factor that contributes to renal injury. Second, the single‐center setting limits generalizability. In addition, convenience sampling may have introduced selection bias and may have contributed to the high CKD prevalence and marked imbalance between the CKD and non‐CKD groups. Third, we did not assess key determinants of HCY such as dietary intake or vitamin B12 and folate status, metformin exposure, smoking, alcohol intake, or dietary protein intake; therefore, residual confounding cannot be excluded. Nor did we conduct longitudinal follow‐up to evaluate the predictive value of HCY for CKD progression over time. Nonetheless, the relatively large sample size and the use of the 2021 CKD‐EPI creatinine–cystatin C equation enhance the robustness of our findings. Although HCY commonly increases as eGFR declines, whether its utility extends beyond reflecting reduced glomerular filtration remains uncertain. The ROC‐derived cutoff of 11.35 μmol/L also requires external validation in independent, more representative T2DM populations.

Future studies should adopt a cohort design to clarify causality and determine whether elevations in HCY precede declines in eGFR and/or the onset of albuminuria. If a temporal relationship is supported, interventional studies could then evaluate whether lowering HCY through folate, vitamins B6/B12 supplementation, or lifestyle modification affects kidney outcomes. In addition, integrating HCY with other biomarkers such as cystatin C, NGAL, KIM‐1, and inflammatory indices could be evaluated in future prognostic models. Population‐specific HCY thresholds should be established according to demographic and nutritional characteristics (e.g., age, sex, nutritional status, and ethnicity) before clinical implementation.

## 5 Conclusion

In this Vietnamese outpatient cohort with T2DM, CKD was highly prevalent and associated with older age. Serum HCY was higher in patients with CKD, increased with worsening eGFR and albuminuria stage, and correlated positively with creatinine, cystatin C, and UACR while correlating inversely with eGFR. Higher HCY remained associated with CKD after adjustment for age and HbA1c. The stronger correlations with filtration markers than with UACR suggest that HCY may predominantly reflect impaired glomerular filtration in this cohort. HCY showed discrimination between CKD and non‐CKD participants (AUC 0.79), but the multivariable AUC of 0.812 did not significantly exceed the HCY‐only model (DeLong *p* = 0.3249). The 11.35 μmol/L cutoff is exploratory and requires external validation; prospective studies are needed before HCY can be considered for screening or prognostic use.

## Author Contributions

**Loi Ngoc Ho:** conceptualization, methodology, data curation, writing – original draft, writing – review and editing, investigation, project administration, funding acquisition. **Tien Van Tran:** methodology, resources. **Tinh Trung Truong Tran:** methodology, software, formal analysis. **Nhan Trong Le:** data curation, visualization, software. **Loc Huu Quach:** writing – original draft, visualization. **Khanh Minh Thanh:** writing – review and editing, validation. **Hien Ngoc Tran:** writing – original draft, visualization. **Hang Khanh Hoang:** writing – original draft, writing – review and editing. **Ha Hai Tran:** data curation. **Tuan Quoc Le:** conceptualization, methodology, writing – review and editing, writing – original draft, investigation, supervision.

## Funding

This study was supported by the University Medical Center Ho Chi Minh City, Ho Chi Minh City, Vietnam.

## Conflicts of Interest

The authors declare no conflicts of interest.

## Supporting information


**File S1:** Ethics approval letter (English translation), Approval No. 1171/HĐĐĐ‐ĐHYD, dated 23 November 2023.

**File S2:** Ethics approval letter (signed Vietnamese original), Approval No. 1171/HĐĐĐ‐ĐHYD, dated 23 November 2023.

## Data Availability

The datasets generated during and/or analysed during the present study are available from the corresponding author on reasonable request.
