# Supplementary material for: Association Between Serum Homocysteine and Chronic Kidney Disease Severity in Vietnamese Outpatients With Type 2 Diabetes: A Cross‐Sectional Study
Source: Endocrinol Diabetes Metab. 2026 Sep 16;9(5):e70339. doi: 10.1002/edm2.70339 (PMC13579793; doi:10.1002/edm2.70339)
Supplement: Supplementary file 1 [file EDM2-9-e70339-s002.pdf]

ĐẠI HỌC Y DƯỢC TP HỒ CHÍ MINH
HỘI ĐỒNG ĐẠO ĐỨC TRONG NCYSH

CỘNG HÒA XÃ HỘI CHỦ NGHĨA VIỆT NAM
Độc lập – Tự do – Hạnh phúc

Số: 1171 /HĐĐĐ-ĐHYD
V/v chấp thuận các vấn đề đạo đức NCYSH

TP. Hồ Chí Minh, ngày 23 tháng 11 năm 2023

CHẤP THUẬN CỦA HỘI ĐỒNG ĐẠO ĐỨC TRONG NGHIÊN CỨU Y SINH HỌC ĐẠI HỌC Y DƯỢC TP. HỒ CHÍ MINH

IRB-VN01002/IORG0008603/FWA00023448

Căn cứ Nghị quyết số 10/NQ-HĐT ngày 20/10/2020 của Hội đồng trường Đại học Y Dược TP. Hồ Chí Minh về việc ban hành Quy chế Tổ chức và hoạt động của Đại học Y Dược TP. Hồ Chí Minh;

Căn cứ Nghị quyết số 26/NQ-HĐT ngày 20/10/2021 của Hội đồng Trường Đại học Y Dược TP. Hồ Chí Minh về việc điều chỉnh một số nội dung tại Nghị quyết số 10/NQ-HĐT ngày 20/10/2020 của Hội đồng Trường về việc ban hành Quy chế tổ chức và hoạt động của Đại học Y Dược TP. Hồ Chí Minh;

Căn cứ Thông tư 04/TT-BYT ngày 5/3/2020 của Bộ trưởng Bộ Y tế quy định việc thành lập, chức năng, nhiệm vụ và quyền hạn của Hội đồng đạo đức trong nghiên cứu y sinh học;

Căn cứ Quyết định số 3870/QĐ-ĐHYD ngày 6/10/2016 của Hiệu trưởng Đại học Y Dược TP. Hồ Chí Minh về việc ban hành Quy chế tổ chức và hoạt động của Hội đồng đạo đức trong nghiên cứu y sinh học;

Căn cứ Quyết định số 939/QĐ-ĐHYD ngày 20/5/2021 của Hiệu trưởng Đại học Y Dược TP. Hồ Chí Minh về việc thành lập Hội đồng đạo đức trong nghiên cứu y sinh học, nhiệm kỳ 2021 - 2026;

Trên cơ sở xem xét của thường trực Hội đồng Đạo đức trong nghiên cứu y sinh học Đại học Y Dược TP. Hồ Chí Minh ngày 16/11/2023.

Hội đồng đạo đức trong nghiên cứu y sinh học chấp thuận về các khía cạnh đạo đức trong nghiên cứu đối với đề tài:

- Tên đề tài: *Nghiên cứu giá trị nồng độ homocysteine huyết thanh và khoảng trống glycat hóa ở bệnh nhân đái tháo đường mắc bệnh thận mạn*
- Mã số: 23815 - ĐHYD
- Người thực hiện: *Trần Văn Tiến – Nghiên cứu sinh*
- Đơn vị chủ trì: *Đại học Y Dược thành phố Hồ Chí Minh*
- Địa điểm triển khai nghiên cứu: *Bệnh viện Đại học Y Dược thành phố Hồ Chí Minh*
- Thời gian tiến hành nghiên cứu: *từ tháng 12/2023 đến tháng 12/2026*
- Phương thức xét duyệt: *Qui trình đầy đủ*

Ngày chấp thuận: 23/11/2023

Lưu ý: **HĐĐĐ có thể kiểm tra ngẫu nhiên trong thời gian tiến hành nghiên cứu**

KT. HIỆU TRƯỞNG
PHÓ HIỆU TRƯỞNG

ĐẠI HỌC Y DƯỢC THÀNH PHỐ HỒ CHÍ MINH

Nguyễn Hoàng Bắc

TM. HỘI ĐỒNG
CHỦ TỊCH HỘI ĐỒNG

Đỗ Văn Dũng
