# Supplementary material for: Association Between Serum Homocysteine and Chronic Kidney Disease Severity in Vietnamese Outpatients With Type 2 Diabetes: A Cross‐Sectional Study
Source: Endocrinol Diabetes Metab. 2026 Sep 16;9(5):e70339. doi: 10.1002/edm2.70339 (PMC13579793; doi:10.1002/edm2.70339)
Supplement: Supplementary file 2 [file EDM2-9-e70339-s001.docx]

| UNIVERSITY OF MEDICINE AND PHARMACY AT HCMC  **BOARD OF ETHICS IN BIOMEDICAL RESEARCH** | **THE SOCIALIST REPUBLIC OF VIETNAM**  Independence – Freedom – Happiness |
| --- | --- |
| Number: 1171/HĐĐĐ-ĐHYD  Re: Accepted ethical issues of biomedical research | *Ho Chi Minh City, November 23^rd^, 2023* |

**APPROVAL FROM THE BOARD OF ETHICS IN BIOMEDICAL RESEARCH
AT UNIVERSITY OF MEDICINE AND PHARMACY AT HO CHI MINH CITY**

IRB-VN01002/IORG0008603/FWA00023448

Pursuant to the Decision No. 10/NQ-HĐT on October 20, 2020 of the Council of University of Medicine and Pharmacy at Ho Chi Minh City on promulgating the Regulation on Organization and Operation of University of Medicine and Pharmacy;

Pursuant to the Decision No. 26/NQ-HĐT on October 20, 2021 of the Council of University of Medicine and Pharmacy at Ho Chi Minh City on adjustments to certain contents of Decision No. 10/NQ-HĐT on October 20, 2020 of the Council on promulgating the Regulation on Organization and Operation of Ho Chi Minh City University of Medicine and Pharmacy;

Pursuant to the Decision No. 04/TT-BYT on March 5, 2020 of the Minister of Health regulating the establishment, functions, duties, and powers of the Board of Ethics in biomedical research;

Pursuant to the Decision No. 3870/QĐ-ĐHYD on October 6, 2016 of the Rector of University of Medicine and Pharmacy at Ho Chi Minh City on promulgating the Regulation on Organization and Operation of the Board of Ethics in biomedical research;

Pursuant to the Decision No. 939/QĐ-ĐHYD on May 20, 2021 of the Rector of University of Medicine and Pharmacy at Ho Chi Minh City on establishing the Board of Ethics in biomedical research, tenure: 2021-2026;

Based on the review of the permanent Board of Ethics in Biomedical Research at University of Medicine and Pharmacy at Ho Chi Minh City on November 16, 2023.

The Board of Ethics has approved the ethical issues of the following research:

- Research title: *Study of homocysteine concentration and glycation gap values in diabetic patients with chronic kidney disease.*
- Research number: 23815 – ĐHYD
- Principal investigator: Tran Van Tien – PhD student
- Oversight institution: University of Medicine and Pharmacy at Ho Chi Minh City
- Research site: University of Medical Center at Ho Chi Minh City
- Research period: from December 2023 to December 2026
- Review category: Full review

***Effective date of approval: November 23^rd^, 2023***

| **PP. RECTOR OF UNIVERSITY OF MEDICINE AND PHARMACY AT HCMC**  **VICE RECTOR**  (Signed and stamped)  Nguyen Hoang Bac | **ON BEHALF OF THE BOARD OF ETHICS**  **CHAIRMAN OF THE BOARD**  (Signed and stamped)  Do Van Dung |
| --- | --- |

*Note:* **The Board of Ethics may randomly check while conducting the research**.
